# Measuring health-related quality of life in children with cancer living in mainland China: feasibility, reliability and validity of the Chinese mandarin version of PedsQL 4.0 Generic Core Scales and 3.0 Cancer Module

**DOI:** 10.1186/1477-7525-9-103

**Published:** 2011-11-23

**Authors:** Yi Ji, Siyuan Chen, Kai Li, Nong Xiao, Xue Yang, Shan Zheng, Xianmin Xiao

**Affiliations:** 1 Department of Pediatric Surgery, Children's Hospital of Fudan University, Shanghai, 201102, China; 2 Department of Pediatric Healthcare, Children's Hospital of Fudan University, Shanghai, 201102, China; 3 Department of rehabilitation center, Children's Hospital of Chongqing Medical University, Chongqing, 400014, China

## Abstract

**Background:**

The Pediatric Quality of Life Inventory (PedsQL) is widely used instrument to measure pediatric health-related quality of life (HRQOL) for children aged 2 to 18 years. The purpose of the current study was to investigate the feasibility, reliability and validity of the Chinese mandarin version of the PedsQL 4.0 Generic Core Scales and 3.0 Cancer Module in a group of Chinese children with cancer.

**Methods:**

The PedsQL 4.0 Genetic Core Scales and the PedsQL 3.0 Cancer Module were administered to children with cancer (aged 5-18 years) and parents of such children (aged 2-18 years). For comparison, a survey on a demographically group-matched sample of the general population with children (aged 5-18) and parents of children (aged 2-18 years) was conducted with the PedsQL 4.0 Genetic Core Scales.

**Result:**

The minimal mean percentage of missing item responses (except the School Functioning scale) supported the feasibility of the PedsQL 4.0 Generic Core Scales and 3.0 Cancer Module for Chinese children with cancer. Most of the scales showed satisfactory reliability with Cronbach's α of exceeding 0.70, and all scales demonstrated sufficient test-retest reliability. Assessing the clinical validity of the questionnaires, statistically significant difference was found between healthy children and children with cancer, and between children on-treatment versus off-treatment ≥12 months. Positive significant correlations were observed between the scores of the PedsQL 4.0 Generic Core Scale and the PedsQL 3.0 Cancer Module. Exploratory factor analysis demonstrated sufficient factorial validity. Moderate to good agreement was found between child self- and parent proxy-reports.

**Conclusion:**

The findings support the feasibility, reliability and validity of the Chinese Mandarin version of PedsQL 4.0 Generic Core Scales and 3.0 Cancer Module in children with cancer living in mainland China.

## Background

Currently, an increasing number of children with cancer are cured for their diseases in the world, including China. However, common treatments such as surgery, radiotherapy, chemotherapy, whether palliative or curative, can have deleterious side-effects affecting many aspect of the quality of children's lives -- not only physical aspects but also social and emotional aspects [1,2]. Survivors may experience a number of long-term adverse effects from the tumor and its treatment [3-6]. Focusing on the patients' psychosocial and social well-being in addition to their physical health is an essential requirement in accordance with the WHO definition of health and well-being [7].

Health-related quality of life (HRQOL) is a continuous concept influenced by a person's objective assessments of function or health status as well as subjective perceptions of their personal health [8]. It is increasingly acknowledged as an important health outcome measure in clinical trials and health services research and evaluation. However, one challenge in measuring HRQOL in children is that the instrument must account for the continuous physical, emotional, social and cognitive development during childhood and adolescence. Another problem is the questionnaires should provide the required information [9]. Although there are a number of generic HRQOL instruments applicable to children, assessment has relied exclusively on proxy-report [10], or the concordance between self- and proxy-report has been demonstrated to be imperfect [11]. Given the multidimensional impact cancer has on children, it is necessary to find an appropriate instrument to capture and evaluate the HRQOL of these children.

The Pediatric Quality of Life Inventory (PedsQL) is one of the more promising HRQOL measures for children aged 2-18 years [12,13]. The advantages of PedsQL included brevity, availability of age-appropriate version, and parallel for child and parent. The approach is modular, with Generic Core Scales complemented by disease-specific modules, such as the PedsQL Cancer Module [14-19]. The PedsQL 4.0 Generic Core Scales was specifically designed for application in both healthy and patient populations. The PedsQL 3.0 Cancer Module was designed to measure HRQOL dimensions specific to pediatric cancers. Studies with PedsQL indicate that both healthy children and children with cancer aged 5-18 years can self-report their HRQOL [20-22].

As the population of Chinese children nears 300 million, a conservative projection of 45 thousands new cases of pediatric cancer each year can be made [23]. However, limited information is available to understand the HRQOL of children with cancer living in mainland China. The objective of this study was to investigate the feasibility, reliability, and validity of the Chinese mandarin version of the PedsQL 4.0 Generic Core Scales and 3.0 Cancer Module in children with cancer.

## Method

### Participants and Settings

This study was developed in the cities of Shanghai and Chongqing, China. We recruited children with cancer and their parents by means of convenience samples from 2 children's hospitals at the cities. Children aged 5 to 18 years who were diagnosed with cancer were included in this study, and the parents were included if their child was 2 to 18 years old. Children were excluded from this study if they had comorbid disease or major developmental disorders. The sample size needed to procedure medium correlation (0.30) in the examination of discriminant validity was calculated. 154 subjects were needed to take part in the study when we set the type Ⅰ error at 1% and the statistical power at 90%. We estimated that approximately more than 60-70% of participants would agree to participate. Finally, two hundred and seventy five families were approached for the study. Controls were 300 families of healthy children aged 2 to 18 years recruited from schools, with distributional matching to the patients on age and gender.

### Measurement

#### PedsQL 4.0 Generic Core Scales

The 23-item PedsQL 4.0 Generic Core Scales encompasses the essential core domains for pediatric HRQOL measurement: (1) physical functioning (8 items), (2) emotional functioning (5 items), (3) social functioning (5 items), and (4) school functioning (5 items). It comprises of parallel child self-report and parent proxy-report format. Child self-report includes ages 5-7 (young children), 8-12 (children), and 13-18 (teens) years. Patent proxy-report includes ages 2-4 (toddlers), 5-7, 8-12, and 13-18 years. The items for each of the forms are essentially identical, differing in developmentally appropriate language, or first or third person tense. The instructions ask how much of a problem each item has been during the past one month. A 5-point response scale is utilized across child self-report for ages 8-18 and parent proxy-report (0 = never a problem; 1 = almost never a problem; 2 = sometimes a problem; 3 = often a problem; 4 = almost always a problem). The child-report for children aged 5-7 is completed with the help of an interviewer. To increase its ease of use, the rating scale has been reworded and simplified to a 3-point scale (0 = not at all a problem; 2 = sometimes a problem; 4 = a lot of a problem), with each response choice anchored to a happy to sad faces scale. Items are reverse-scored and linearly transformed to a 0-100 scale, with higher scores indicating a better HRQOL. Scale scores are computed as the sum of the items divided by the number of items answered. If more than 50% of the items in the scale are missing, the scale score is not computed. To create the Psychosocial Functioning score, the mean is computed as the sum of the items divided by the number of the items answered in the Emotional, Social, and School Functioning Scale.

#### PedsQL 3.0 Cancer Module

The PedsQL 3.0 Cancer Module instrument encompasses 8 subscales: (1) pain and hurt (2 items), (2) nausea (5 items), (3) procedural anxiety (3 items), (4) treatment anxiety (3 items), (5) worry (3 items), (6) cognitive problems (5 items), (7) perceived physical appearance (3 items), and (8) communication (3 items). The cognitive problems scales were modified to include fewer items for children aged 2-7 years. The format, instructions, Likert response scale, and scoring method of the Cancer Module are identical to the PedsQL 4.0 Generic Core Scales [24].

### Procedure

The investigation was performed by 4 research students majoring in Pediatrics and 2 nurses. All of them were trained in interviewing and administering questionnaires. On-treatment status was defined as patients who were receiving medical treatment to induce remission. Off-treatment status was defined as patients who completed all therapy by the time of the assessment [25,26]. The on-treatment patient was interviewed while hospitalized. The off-treatment patient was interviewed during clinic visits. Parents were interviewed first, and were asked whether they would allow their children to participate in the study. The parents and their children completed the questionnaire independently during the pediatric patients' hospitalization or outpatient department visit. All the parents were asked to fill out the PedsQL 4.0 Generic Core Scales and the Cancer Module. The children were required to complete the questionnaires by self-administration except the Young Children by interview-administration [19]. The interviewers were available to assist the completion of the questionnaires if the parents/children had questions on semantic or conceptual understanding. They were also responsible for collecting and checking the questionnaires to ensure that there were no missing data or logical mistakes. Test-retest reliability was assessed at Children's Hospital of Fudan University (the same hospital in Shanghai). Thirty-four families with patients in stable condition according to their physician completed the PedsQL measures a second time (with an interval of 1-3 weeks between applications). For control group, the questionnaires interviews were conducted at schools. Written information was sent to parents who completed questionnaires at home, returning them to school by a specified date.

### Cross-culture adaptation and Ethical considerations

The Chinese Mandarin Version was provided by MAPI Research Trust that translated the version according to their standardized procedures. Briefly, the instruments were translated independently into Chinese by two professional translators (native Chinese speakers) and translated back into English by two English native speakers (bilingual in Chinese). Then, a comparison between the backward version and the original version was assessed in order to detect any misunderstandings or mistranslations in the intermediary forward version of the questionnaires [27,28]. 15 children with cancer participated in pilot testing along with their parents. In consideration of the Chinese sociocultural environment, we avoided using the term 'cancer' or 'tumor' in questionnaires. The permission was obtained from its developer, James W. Varni, PhD. The human subject institutional review boards at each hospital approved the study. All subjects were given detailed written information about the methods, aims, and the voluntary nature of participation in the study, and written parental informed consent and child assent were obtained prior to enrollment.

### Statistical analysis

Feasibility was determined from the average completion time and percentage of missing response. The average completion time was defined as the mean of completion time of the Generic Core Scales and Cancer Module. The percentage of all possible item-responses left unanswered was calculated for each subject on each single and summary scale and averaged over subjects [29]. The presence of floor and ceiling effects was assessed for the subscales scores and summary scores.

Scale internal consistency reliability was determined by calculating Cronbach's coefficient alpha. Scales with reliabilities of 0.70 or greater are recommended for comparing patients groups, while a reliability criterion of 0.90 is recommended for analyzing individual patient scale scores [30]. To determine retest reliability, the intra-class correlation coefficients (ICCs) between the initial test and retest scores was examined, with an ICC value of 0.40 representing moderate, 0.60 good, and 0.80 high agreement [31].

Discriminant validity was determined using the known-groups method. The Generic Core Scales scores were compared between groups differing in known health conditions (healthy children and children with cancer). HRQOL scores of children from the general population and children with cancer were compared using independent sample *t *test. To determine the magnitude of the differences, effect sizes were evaluated. Effect size as utilized in these analyses was calculated by taking the difference between the healthy sample mean and the oncology sample mean, divided by the pooled standard deviation [32]. Additionally, analyses of variance also were conducted to examine whether there were differences in Generic Core Scales and Cancer Module scores among children with cancer on treatment and off-treatment (≤ 12 months or > 12 months). We hypothesized that healthy children would have higher PedsQL 4.0 scores (better HRQOL) than children with cancer. Moreover, we hypothesized that children with cancer on-treatment would report significant differences in HRQOL compared with those of children with cancer off-treatment based on previous studies [19,33,34].

Construct validity for the Cancer Module was examined through an analysis between the Cancer Module Scales scores and relevant Generic Core Scales scores. It had been reported that computing the intercorrelations among scales provides initial information on the construct validity of an instrument. We hypothesized greater disease-specific symptoms or problems would correlate with lower overall generic HRQOL based on the conceptualization of disease-specific symptoms as causal indicators of generic HRQOL [35]. Correlation effect sizes were designed as small (0.01-0.29), medium (0.30-0.49), and large (≥ 0.50). Pearson coefficients were utilized in these analyses.

Exploratory factor analysis was performed on the items to test the PedsQL underlying dimensions [36]. Principal component analysis with oblique rotation was performed to extract the factors. Factors with an eigenvalue less than 1.0 were disregarded.

Parent/child intercorrelations were computed to examine cross-informant variance [37]. Correlation effect sizes are designated as small (0.01-0.29), medium (0.30-0.49), and large (≥ 0.50).

Statistical analyses of the study were conducted by SPSS 16.0 for Window (SPSS, Inc, Chicago, USA) and the significance level was set at 0.05.

## Results

### Sample characteristics

Of the patients group, 7 families refused to participate due to non-compliance, 2 children were later found to be ineligible and excluded from analysis, leaving 266 eligible families. The patients group was comprised of 202 children aged 5-18 years and 266 parents of children aged 2-18 years. The mean age of the 154 boys (57.9%) and 112 girls (42.1%) was 8.8 years (SD = 3.8). One hundred sixty-three patients (61.3%) had been diagnosed with hematological diseases, and the remaining patients (38.7%) had solid tumors. The mother answered the questionnaires in 82.7% of the cases and the father in 17.3% of the cases. 109 (41.0%) patients completed all therapy by the time of assessment (n = 109), and 74 (27.8%) patients had been off treatment for over 12 months (n = 74). Of the control group, 284 families returned the questionnaires, which gave a response rate of 94.7%. The mean age of the 159 boys (56.0%) and 125 girls (44.0%) was 9.1 years (SD = 3.9). Self-report forms are completed by 216 children (Table 1). There was no statistically significant difference between patients and healthy children refer to age and gender.

**Table 1:** Characteristics of the patients with cancer and healthy children

Variables	Patients with cancer									
	Child On-treatment (n = 157)		Child off-treatment < 12 m (n = 35)		Child off-treatment > 12 m (n = 74)		Total sample (n = 266)		Healthy Children (n = 284)	
	n	%	n	%	n	%	n	%	n	%
Age										
2-4	47	29.9	9	25.7	8	10.8	64	24.1	68	23.9
5-7	42	26.8	10	28.6	25	33.8	77	28.9	81	28.5
8-12	38	24.2	8	22.9	23	31.1	69	25.9	74	26.1
13-18	30	19.1	8	22.9	18	24.3	56	21.1	61	21.5
Gender†										
Male	92	58.6	20	57.1	42	56.8	154	57.9	159	56.0
Female	65	41.4	15	42.9	32	43.2	112	42.1	125	44.0
Diagnosis										
Leukemia or lymphoma	91	58.0	22	62.9	50	67.6	163	61.3	-	
Solid tumor	66	42.0	13	37.1	24	32.4	103	38.7	-	
Education										
Non-attendance	50	31.8	9	25.7	12	16.2	71	26.7	0	0.0
Current school attendance	107	68.2	26	74.3	62	83.8	195	73.3	284	100.0
Parents										
Mother	131	83.4	27	77.1	62	83.8	220	82.7	237	83.5
Father	26	16.6	8	22.9	12	16.2	46	17.3	47	16.5

n: number of individuals.

### Descriptive statistics

As showed in Table 2 the Generic Core scores were consistently higher for child reports than for parent reports. No floor effects were seen in the patient group. We found ceiling effects both in child self- and parent proxy-reports ranging from 2.6% to 19.8% in the patient group and 5.6 to 39.8% in the control group, with highest values in the Social Functioning Scale for child self- and parent proxy-reports from the patient and comparison samples. We also observed greater ceiling (18.0-40.6%) than floor effects (0.8-5.6%) in the Cancer Module, with a notable ceiling effect in the Pain and hurt scale and a moderate one in other scales for child self- and parent proxy-reports (Table 3).

**Table 2:** Scale descriptives for PedsQL 4.0 Generic Core Scales child self- and parent proxy-report, and comparisons between children with cancer and healthy children scores

Scale	Children with cancer			Healthy children			Effect size*	*t*†
	n	Mean (SD)	%Floor/%Ceiling	n	Mean (SD)	%Floor/%Ceiling		
Child Self-report								
Total score	202	68.56 (17.4)	0.00/4.0	216	84.72 (16.4)	0.00/6.5	0.96	9.77
Physical functioning	202	67.96 (19.9)	0.00/11.4	216	86.33 (16.1)	0.00/18.5	1.03	10.39
Psychosocial functioning	202	69.20 (18.4)	0.00/5.1	216	84.20 (17.3)	0.00/16.2	0.86	8.59
Emotional functioning	202	68.40 (20.9)	0.00/12.9	215	80.25 (15.8)	0.00/37.2	0.68	6.57
Social functioning	202	78.31 (16.1)	0.00/19.8	216	88.16 (17.6)	0.00/39.8	0.57	5.95
School functioning	165	62.19 (22.5)	0.00/8.5	213	83.81 (16.2)	0.00/31.9	1.15	10.87
Parent Proxy-report								
Total score	266	65.54 (18.8)	0.00/2.6	284	80.74 (16.9)	0.00/5.6	0.87	9.81
Physical functioning	266	66.13 (20.5)	0.38/10.9	284	82.18 (17.6)	0.00/15.8	0.92	9.88
Psychosocial functioning	266	65.06 (18.7)	0.00/4.2	284	80.09 (17.6)	0.00/15.1	0.84	9.69
Emotional functioning	266	64.07 (19.5)	0.00/11.3	284	76.90 (16.6)	0.00/33.8	0.73	8.32
Social functioning	266	73.49 (17.6)	0.00/18.8	284	85.33 (15.4)	0.00/38.0	0.70	8.42
School functioning	195	60.55 (23.6)	0.00/6.7	284	78.03 (16.5)	0.00/29.9	0.87	9.54

SD: standard deviation.

*Effect size for differences in means are designated as small (0.20-0.49), medium (0.50-0.79), and large (0.80 and above) in magnitude.

†All scale scores for the children with caner are significant differences from healthy children (*p *< .0.01) based on the independent sample *t *test.

**Table 3:** Scale descriptives of the PedsQL 3.0 Cancer Module for child self- and parent proxy-reports

Scale	Child self-reports			Parent proxy-reports		
	n	Mean (SD)	%Floor/%Ceiling	n	Mean (SD)	%Floor/%Ceiling
Pain and hurt	202	74.24 (21.89)	1.0/40.6	266	77.41 (20.72)	0.8/38.0
Nausea	202	75.73 (20.54)	1.5/37.1	266	73.21 (19.45)	1.9/33.8
Procedural anxiety	202	68.02 (27.34)	4.5/28.2	266	64.14 (24.73)	5.6/20.8
Treatment anxiety	202	71.68 (24.24)	4.0/26.7	266	67.13 (23.69)	3.0/20.1
Worry	201	68.14 (25.19)	3.0/31.6	262	62.92 (24.30)	1.9/23.2
Cognitive problems	200	71.72 (29.33)	3.0/18.5	260	73.93 (26.60)	1.2/18.0
Perceived physical appearance	202	76.35 (20.47)	3.5/33.8	261	77.91 (17.21)	0.8/28.0
Communication	201	72.19 (20.19)	1.5/24.7	262	71.63 (23.61)	1.5/25.0

### Feasibility

The majority of patients and their parents needed only 10-18 and 6-10 minutes to complete the two questionnaires, respectively. For child self- and parent proxy-report on the PedsQL 4.0 Generic Core Scales, the percentage of missing item responses was 0.6% and 0.4%, respectively, for all scales except the School Functioning Scale. The percentage of missing items for the School Functioning scale was 21.5% for child self-report (aged 5-18 years) and 46.3% for parent proxy-report (aged 2-18 years). This large percentage missing items for the School Functioning scale may exist because many Chinese children younger than 7 years neither attend kindergarten nor school. For child self- and parent proxy-report on the PedsQL 3.0 Cancer Module, the percentage of missing item responses was 0.9% and 1.3%, respectively, for all scales. On this module, 40.7% of the items across all forms had no missing responses. The highest percentage of missing responses for any single item on the Caner Module was 3.2% for Child self-report in the Communication Ⅱ scale (it is had for me to ask the doctors and nurses questions) and 5.9% for parent proxy-report in the WorryⅠ scale (worry about side effect from medical treatment).

### Internal consistency and test-retest reliability

Internal consistency reliability coefficients are presented in Table 4. Cronbach's alpha for Total Scale of the Generic Scales and the Caner Scale were all above 0.7 in both self- and proxy-reports. Most scales exceeded the minimum reliability standard of 0.70, and a number of scales approached or met the reliability criterion of 0.90 recommended for analyzing individual patient scores.

**Table 4:** Reliability of the Chinese mandarin version of the PedsQL 4.0 Generic Core Scales and 3.0 Cancer Module: self- and proxy-reports

Scale	Internal consistency reliability										Retest reliability
	2-4 years		5-7 years		8-12 years		13-18 years		Total		
	n	α	n	α	n	α	n	α	n	α	ICC
Generic Scale											
Total score	NA		77	0.82	69	0.85	56	0.89	202	0.86	0.81
	**64**	**0.87**	**77**	**0.90**	**69**	**0.92**	**56**	**0.91**	**266**	**0.91**	**0.83**
Physical functioning	NA		77	0.65	69	0.83	56	0.82	202	0.81	0.90
	**64**	**0.84**	**77**	**0.88**	**69**	**0.85**	**56**	**0.86**	**266**	**0.84**	**0.89**
Psychosocial functioning	NA		77	0.72	69	0.84	56	0.81	202	0.82	0.79
	**64**	**0.85**	**77**	**0.87**	**69**	**0.88**	**56**	**0.87**	**266**	**0.86**	**0.78**
Emotional functioning	NA		77	0.68	69	0.78	56	0.76	202	0.73	0.74
	**64**	**0.72**	**77**	**0.73**	**69**	**0.76**	**56**	**0.80**	**266**	**0.79**	**0.77**
Social functioning	NA		77	0.56	69	0.68	56	0.79	202	0.71	0.71
	**64**	**0.76**	**77**	**0.82**	**69**	**0.79**	**56**	**0.83**	**266**	**0.80**	**0.75**
School functioning	NA		49	0.47	64	0.66	55	0.69	165	0.65	0.78
	**27**	**0.68**	**49**	**0.75**	**64**	**0.72**	**55**	**0.71**	**195**	**0.72**	**0.84**
Cancer scale											
Total scale	NA		77	0.70	69	0.79	56	0.81	202	0.77	0.76
	**64**	**0.83**	**77**	**0.85**	**69**	**0.87**	**56**	**0.88**	**266**	**0.87**	**0.84**
Pain and hurt	NA		77	0.66	69	0.70	56	0.72	202	0.69	0.61
	**64**	**0.80**	**77**	**0.83**	**69**	**0.87**	**56**	**0.90**	**266**	**0.88**	**0.75**
Nausea	NA		77	0.78	69	0.80	56	0.85	202	0.81	0.63
	**64**	**0.83**	**77**	**0.83**	**69**	**0.88**	**56**	**0.86**	**266**	**0.85**	**0.79**
Procedural anxiety	NA		77	0.74	69	0.82	56	0.84	202	0.76	0.77
	**64**	**0.85**	**77**	**0.86**	**69**	**0.89**	**56**	**0.88**	**266**	**0.89**	**0.73**
Treatment anxiety	NA		77	0.75	59	0.81	56	0.83	202	0.78	0.75
	**64**	**0.88**	**77**	**0.87**	**59**	**0.87**	**56**	**0.89**	**266**	**0.88**	**0.61**
Worry	NA		76	0.55	59	0.76	56	0.81	201	0.75	0.82
	**61**	**0.90**	**76**	**0.86**	**59**	**0.85**	**56**	**0.88**	**262**	**0.87**	**0.79**
Cognitive problems	NA		75	0.50	59	0.70	56	0.74	200	0.74	0.68
	**59**	**0.71**	**76**	**0.79**	**59**	**0.78**	**56**	**0.87**	**260**	**0.80**	**0.80**
Perceived physical appearance	NA		77	0.54	59	0.65	56	0.71	201	0.61	0.74
	**60**	**0.62**	**76**	**0.71**	**59**	**0.74**	**56**	**0.80**	**261**	**0.75**	**0.80**
Communication	NA		76	0.62	59	0.70	56	0.73	202	0.69	0.72
	**60**	**0.87**	**77**	**0.82**	**59**	**0.84**	**56**	**0.81**	**262**	**0.84**	**0.70**

Reliability of parent proxy-report is set in boldface.

NA: not applicable; α: Cronbach's coefficient alpha; ICC: intraclass correlation coefficient.

Retests for reliability were completed by 27 children with cancer (aged 5-18 years) and 34 patents of such children (aged 2-18 years) who completed the initial questionnaires. ICCs for test-retest reliability for child self- and parent proxy-report are presented in Table 4. All of these ICCs are in the good to excellent reliability range.

### Validity

Table 2 demonstrates the differences between healthy children and children with cancer. For each Generic Core Scale, children with cancer and their parents report statistically significant lower HRQOL than healthy children. Table 5 provides the result comparing the three groups of patients in known distinct clinical conditions (on-treatment, off treatment ≤ 12 months and off-treatment > 12 months) for child self- and parent proxy-report on the PedsQL Generic Core Scales and Cancer Module. For both child self- and parent proxy-report, the PedsQL 4.0 Generic Core Scale Total Score, Physical Functioning, and Emotional Functioning scores demonstrated significant differences between the patients on-treatment and off-treatment > 12 months. For the PedsQL 3.0 Caner Module Scales, children who had been off-treatment over 12 months and their parents demonstrated significant higher scores than children who had been on-treatment on the Pain and hurt, Nausea, and Procedural Anxiety subscales. In addition, the scores of parent proxy-report Treatment Anxiety and Worry subscale were significant differences between children on-treatment versus off-treatment > 12 months.

**Table 5:** One-way ANOVA comparing HRQOL (generic scale and cancer scale) between children on- and off-treatment (≤ 12 months or > 12 months): self- and proxy-report (bold)

Scales	Child On-treatment (a)		Child off-treatment (b)		Child off-treatment (c)		Difference	F	*P*
			≤ 12 months		> 12 months				Value
	n	mean (SD)	n	Mean (SD)	n	Mean (SD)			
Generic Scale									
Total score	110	65.42 (16.98)	26	67.14 (18.05)	66	73.69 (16.19)	a < c**	5.04	0.007
	**157**	**62.33 (16.70)**	**35**	**65.92 (19.43)**	**74**	**71.22 (17.92)**	a < c***	**6.58**	**0.002**
Physical functioning	110	62.57 (21.08)	26	67.12 (18.25)	66	76.26 (19.24)	a < c***, b < c*	9.53	0.000
	**157**	**61.55 (18.67)**	**35**	**66.18 (20.63)**	**74**	**75.51 (21.15)**	a < c***, b < c*	12.7	**0.000**
Psychosocial functioning	110	67.94 (17.53)	26	67.23 (19.77)	66	72.20 (18.92)		1.30	0.275
	**157**	**63.07 (19.40)**	**35**	**66.04 (17.36)**	**74**	**68.87 (18.56)**		**2.42**	**0.091**
Emotional functioning	110	64.71 (19.54)	26	67.36 (22.81)	66	73.64 (20.16)	a < c**	4.05	0.019
	**157**	**59.82 (24.58)**	**35**	**68.40 (21.82)**	**74**	**70.71 (18.44)**	a < b*, a < c***	6.44	0.002
Social functioning	110	77.97 (17.48)	26	75.03 (18.24)	66	79.30 (15.68)		0.59	0.556
	**157**	**72.02 (20.68)**	**35**	**72.26 (18.52)**	**74**	**75.16 (18.79)**		**0.65**	**0.524**
School functioning	86	58.47 (19.89)	20	60.35 (18.55)	59	65.18 (23.09)		1.81	0.167
	**107**	**57.96 (19.30)**	**26**	**60.80 (22.25)**	**62**	**62.94 (24.35)**		**1.08**	**0.342**
Cancer scale									
Pain and hurt	110	70.23 (20.54)	26	75.35 (20.19)	66	78.66 (22.47)	a < c*	3.38	0.036
	**157**	**74.68 (19.03)**	**35**	**77.81 (21.45)**	**74**	**82.12 (20.60)**	a < c**	3.57	**0.029**
Nausea	110	63.92 (22.36)	26	78.72 (19.11)	66	83.20 (18.59)	a < b**, a < c***	19.22	0.000
	**157**	**66.81 (18.29)**	**35**	**72.34 (23.05)**	**74**	**81.72 (16.14)**	a < c***, b < c*	**16.5**	**0.000**
Procedural anxiety	110	63.17 (28.31)	26	70.29(25.25)	66	73.33 (24.90)	a < c*	3.12	0.046
	**157**	**62.42 (22.63)**	**35**	**64.71 (25.39)**	**74**	**76.26 (24.87)**	a < c***, b < c*	**8.74**	**0.000**
Treatment anxiety	110	68.77 (23.48)	26	72.11 (26.23)	66	73.84 (20.64)		1.05	0.352
	**157**	**62.85 (20.69)**	**35**	**68.05 (24.38)**	**74**	**72.97 (23.38)**	a < c**	**5.46**	**0.005**
Worry	110	66.59 (23.24)	26	70.33 (28.10)	66	71.32 (25.61)		0.83	0.438
	**155**	**59.13 (22.00)**	**35**	**60.78 (24.43)**	**72**	**67.20 (24.74)**	a < c*	**3.06**	**0.049**
Cognitive problems	108	70.09 (25.65)	26	73.83 (30.02)	66	71.75 (29.45)		0.22	0.804
	**154**	**72.11 (23.96)**	**34**	**74.44 (29.12)**	**72**	**74.25 (26.92)**		**0.23**	**0.791**
Perceived physical appearance	110	74.85 (19.48)	26	76.98 (22.17)	66	79.65 (18.34)		1.24	0.291
	**154**	**77.21 (16.90)**	34	74.93 (20.55)	**73**	**75.77 (17.49)**		0.32	**0.723**
Communication	110	72.66 (19.11)	26	73.27 (18.40)	65	70.98 (22.75)		0.18	0.832
	**155**	**71.18 (22.83)**	**35**	**75.29 (24.52)**	**72**	**72.04 (22.38)**		**0.46**	**0.633**

*P < 0.05, **P < 0.01, ***P < 0.001 based on Tukey Honestly Significantly Different post hoc analysis.

The result of the factor analysis for child self- and parent proxy-report of the Generic Core Scales and Cancer Module are presented in Table 6 and 7. For the Generic Core Scales, an eigenvalue cutoff of 1.0 resulted in a six factor solution for child self- and parent proxy-report, accounting for 62.3% and 69.6% of the variance, respectively. For the Cancer Module, an eight factor solution for child self-report was result, accounting for 78.0% of the variance; and a seven-factor solution for parent proxy-report was result, accounting for 86.6% of the variance.

**Table 6:** PedsQL 4.0 Generic Core Scales factor loadings for child self- and parent proxy-report in children with cancer

Subscale	Item	Factor 1	Factor 2	Factor 3	Factor 4	Factor 5	Factor 6
Physical functioning	P1	**.666**	.000	.153	.065	.131	.126
		*.338*	***.700***	*.078*	*.041*	*.064*	*.005*
	P2	**.825**	-.037	.127	.101	-.021	.123
		*-.031*	***.779***	*.250*	*.092*	*.121*	*.243*
	P3	**.828**	.047	-.029	-.135	.100	-.022
		*-.020*	***.756***	*-.093*	*.168*	*.025*	*.042*
	P4	**.804**	.274	-.052	-.249	.004	.036
		*-.123*	***.807***	*-.116*	*.157*	*.210*	*.062*
	P5	**.721**	.468	.091	.241	-.024	-.034
		*.185*	***.693***	*.153*	*-.046*	*.190*	*.151*
	P6	.373	-.002	.203	**.477**	.094	.051
		*-.043*	***.658***	*.292*	*.082*	*.256*	*.074*
	P7	**.620**	.139	-.103	.172	.023	.072
		***.620***	*.345*	*.055*	*-.189*	*.028*	*.186*
	P8	**.527**	.228	.059	.121	.114	-.157
		***.571***	*.073*	*.108*	*.183*	*.259*	*.068*
Emotional functioning	E1	-.018	.070	**.830**	.165	.082	.130
		*-.049*	*.225*	*-.129*	*.048*	***.777***	*.009*
	E2	.026	.188	**.710**	.026	.102	.035
		*.093*	*.225*	*-.021*	*.179*	***.780***	*-.163*
	E3	.136	.217	**.688**	.105	.135	.121
		*.225*	*.182*	*.012*	*.007*	***.748***	*.134*
	E4	-.005	.002	.263	-.013	.071	**.755**
		*.205*	*.148*	*.050*	***.726***	*.377*	*-.070*
	E5	.173	-.206	.305	-.048	-.084	**.638**
		*.200*	*.143*	*-.082*	***.555***	*.407*	*-.268*
Social functioning	S1	.227	**.697**	.209	.179	-.302	.199
		*.202*	*.152*	***.700***	*.293*	*-.158*	*-.128*
	S2	.160	**.848**	.005	.009	.069	.178
		*-.026*	*-.017*	*.367*	***.777***	*.058*	*.124*
	S3	-.025	**.695**	.217	.126	.216	-.271
		*-.155*	*.040*	***.563***	*.217*	*.232*	*.176*
	S4	.199	.412	.781	.256	-.064	.009
		*-.203*	*.039*	***.506***	*.089*	*.268*	*.074*
	S5	.025	**.668**	.265	.318	.198	.131
		*.017*	*.254*	***.756***	*.092*	*.044*	*.003*
School functioning	Sc1	-.207	.378	.006	**.716**	-.059	.069
		*.161*	*.078*	*.134*	*-.102*	*.138*	***.664***
	Sc2	-.038	.031	.255	**.802**	.021	-.170
		*-.040*	*-.014*	*.187*	*.189*	*.039*	***.521***
	Sc3	-.068	.187	.089	**.600**	.194	.072
		*.179*	*.041*	*.181*	*.052*	*.047*	*.833*
	Sc4	.154	.096	.021	.040	**.923**	-.094
		***.860***	*.135*	*-.045*	*.181*	*-.012*	*.290*
	Sc5	.030	.044	.253	.095	**.908**	.152
		*.111*	*.233*	*.015*	*-.119*	.110	***.813***
Eigenvalue		6.744	2.844	2.208	1.878	1.579	1.145
		*7.457*	*3.554*	*2.453*	*1.956*	*1.699*	*1.182*
Percent Variance		25.32	10.364	9.602	7.164	5.865	3.977
		*28.074*	*11.103*	*10.665*	*8.071*	*6.517*	*5.138*

Note: Bold values indicate the largest factor loading for each item.

In each cell, child self-report loading are shown above and the parent proxy-report loading are shown below in italics.

Extraction Method: Principle Component Analysis. Rotation Method: Oblimin with Kaiser Normalization. Total Variance Explained for child self-report: 62.3%; for Parent Proxy-report: 69.6%.

**Table 7:** PedsQL 3.0 Cancer Module factor loadings for child self- and parent proxy-report in children with cancer

Subscale	Item	Factor 1	Factor 2	Factor 3	Factor 4	Factor 5	Factor 6	Factor 7	Factor 8
Pain and hurt	P1	.034	.104	.008	.023	**.689**	.150	.071	.104
		*-.009*	*.023*	*.029*	*-.16*	*.066*	*.009*	***.533***	
	P2	.136	.206	.072	.049	**.714**	-.171	.031	.073
		*.097*	*.075*	*.017*	*.180*	*.091*	*.056*	***.789***	
Nausea	N1	**.854**	.115	.121	.039	-.06	-.173	-.123	.219
		*.158*	***.835***	*-.057*	*.087*	*-.086*	*-.079*	*.016*	
	N2	**.657**	.028	-.094	.044	.253	.005	.007	.130
		*.004*	***.573***	*.080*	*-.038*	*.021*	*.022*	*-.046*	
	N3	**.789**	.018	.082	-.009	.107	-.094	-.02	-.007
		*.199*	***.827***	*-.013*	*.156*	*-.057*	*.069*	*.134*	
	N4	**.668**	-.183	.038	.019	.032	.111	-.04	-.072
		*.052*	***.731***	*-.123*	*.070*	*.075*	*.147*	*.343*	
	N5	**.821**	-.008	.145	.114	.049	.138	-.077	-.033
		*.190*	***.727***	*.089*	*.026*	*.244*	*.080*	*.093*	
Procedural anxiety	PA1	.011	.159	.042	**.866**	.061	.103	-.133	.199
		***.766***	*-.114*	*.043*	*-.061*	*.070*	*-.047*	*.184*	
	PA2	.050	.043	-.03	**.876**	.055	-.100	.035	-.08
		***.860***	*.156*	*.030*	*-.022*	*-.156*	*.070*	*.361*	
	PA3	-.057	.137	.212	**.917**	.011	-.069	.099	.061
		***.848***	*.035*	*-.044*	*-.095*	-.007	.062	.005	
Treatment anxiety	TA1	.350	**.840**	.040	.158	.135	.009	.133	-.1
		***.821***	*.024*	*.085*	*.248*	*.114*	*.105*	*.045*	
	TA2	.071	**.795**	.127	.030	.135	.069	.193	-.068
		***.864***	*.290*	*.092*	*.096*	*.210*	*.157*	*-.082*	
	TA3	.156	**.861**	.011	.186	.293	-.101	-.021	-.035
		***.888***	*.155*	*.116*	*.245*	*.046*	*.041*	-.099	
Worry	W1	.049	.013	**.622**	-.011	.127	.028	.049	-.095
		*.033*	*.043*	*.118*	***.561***	*.040*	*.060*	*.063*	
	W2	.253	.172	**.751**	.176	.033	.044	.083	.003
		*.021*	*.024*	*.014*	***.775***	*.142*	*-.022*	*.191*	
	W3	.066	.021	**.807**	.125	.004	.058	.062	.201
		*-.054*	*.099*	*.078*	***.775***	*.020*	*.184*	*.004*	
Cognitive problems	CP1	-.197	-.03	.012	.120	.031	**.882**	.105	.038
		*.196*	*.137*	***.849***	*-.051*	*.273*	*.087*	*.163*	
	CP2	.004	-.165	-.04	-.034	-.083	**.863**	.079	.138
		*.112*	*.051*	***.9***	*.034*	*.070*	*.219*	*.073*	
	CP3	-.137	-.041	-.198	.011	.391	**.631**	.120	-.009
		*-.013*	*-.004*	***.614***	*.191*	*.243*	*.091*	*.172*	
	CP4	-.002	.221	.006	-.119	.085	**.706**	.098	.109
		*.223*	*-.044*	***.742***	*.002*	*.180*	*.279*	*-.057*	
	CP5	-.204	.134	-.109	-.073	.177	**.665**	.074	-.022
		*.061*	*.085*	***.794***	*.155*	*.176*	*-.053*	*-.16*	
Perceived physical appearance	A1	.005	**.820**	-.14	.050	-.087	-.024	-.192	.076
		*.041*	*.161*	*.246*	*.088*	*.112*	***.767***	*.270*	
	A2	.024	**.757**	.046	.050	-.215	-.073	-.094	.013
		*.060*	*-.094*	*-.162*	*.179*	*.212*	***.637***	*-.062*	
	A3	.213	**.857**	.166	-.198	-.18	.036	.033	-.047
		*-.079*	*-.106*	*.030*	*.142*	*.079*	***.666***	*-.03*	
Communication	C1	.123	.101	.182	.118	.159	.020	.060	**.611**
		*.009*	*.131*	*.074*	*.044*	***.843***	*.311*	*-.016*	
	C2	-.083	-.107	.078	.040	.148	.1	-.041	**.905**
		*.024*	*.181*	*.193*	*.175*	***.551***	*-.148*	*.046*	
	C3	.010	-.005	.281	.098	.048	.035	-.188	**.742**
		*.113*	*-.144*	*.110*	*.040*	***.743***	*.105*	*.061*	
Eigenvalue		6.548	4.303	3.635	2.319	2.074	1.771	1.34	1.096
		*8.682*	*5.225*	*3.725*	*2.756*	*2.142*	*1.843*	*1.447*	
Percent Variance		24.253	13.936	9.462	7.587	7.182	6.56	4.962	4.057
		*32.157*	*15.647*	*1.091*	*8.613*	*8.081*	*6.715*	*5.246*	

Note: Bold values indicate the largest factor loading for each item.

In each cell, child self-report loading are shown above and the parent proxy-report loading are shown below in italics.

Extraction Method: Principle Component Analysis. Rotation Method: Oblimin with Kaiser Normalization. Total Variance Explained for child self-report: 78.0%; for Parent Proxy-report: 86.6%.

As to the intercorrelations among the various Generic Core Scales and the Cancer Module scales estimated using correlation coefficients. As anticipated, correlation coefficients between the Generic Core Scale Total Scale and the Cancer Module subscales were of medium to large effect size for both the child self- and parent proxy-reports (Table 8).

**Table 8:** Intercorrelations among PedsQL Scales and correlation between scores of the child and parent

	Tot	PH	Psy	Em	Soc	Sch	P	N	PA	TA	W	CP	A	C
Total Score (Tot)	0.493**	0.826**	0.853**	0.818**	0.744**	0.620**	0.476**	0.428**	0.434**	0.380**	0.395**	0.336**	0.304**	0.318**
Physical functioning (Ph)	0.878**	0.501**	0.591**	0.506**	0.530**	0.486**	0.429**	0.278**	0.316**	0.280**	0.303**	0.245**	0.266**	0.271**
Psychosocial functioning (Psy)	0.905**	0.585**	0.336**	0.647**	0.631**	0.677**	0.298**	0.322**	0.303**	0.254**	0.370**	0.333**	0.395**	0.257**
Emotional functioning (Em)	0.860**	0.494**	0.848**	0.327**	0.507**	0.293**	0.231**	0.210**	0.287**	0.224**	0.266**	0.205**	0.239**	0.194*
Social functioning (Soc)	0.798**	0.535**	0.869**	0.605**	0.359*	0.413**	0.203**	0.136	0.206**	0.264**	0.277**	0.248**	0.233**	0.220**
School functioning (Sch)	0.729**	0.497**	0.785**	0.375**	0.523**	0.305**	0.356**	0.229**	0.274**	0.297**	0.283**	0.394**	0.195**	0.118
Pain and hurt (P)	0.513**	0.447**	0.461**	0.413**	0.386**	0.342**	0.442**	0.228**	0.297**	0.273**	0.465**	0.305**	0.341**	0.282**
Nausea (N)	0.479**	0.302**	0.337**	0.314**	0.291**	0.292**	0.220**	0.406**	0.235*	0.365**	0.353**	0.238*	0.174*	0.120
Procedural anxiety (PA)	0.365**	0.278**	0.285**	0.290**	0.272**	0.228**	0252**	0.032	0.349**	0.483**	0.377**	0.174*	0.319**	0.230**
Treatment anxiety (TA)	0.326**	0.298**	0.382**	0.324**	0.273**	0.204**	0.189*	0.239**	0.434**	0.420*	0.329**	0.341**	0.260**	0.285**
Worry (W)	0.396**	0.237**	0.410**	0.228**	0.239**	0.210**	0.281**	0.281*	0.203**	0.371**	0.354**	0.214**	0.357**	0.219**
Cognitive problems CP)	0.348**	0.365**	0.376**	0.253**	0.327**	0.424**	0.086	0.139	0.251**	0.349**	0.311**	0.308*	0.398**	0.276**
Appearance (A)	0.315**	0.249**	0.329**	0.295**	0.285**	0.219**	0.253**	0.182*	0.184*	0.335**	0.274**	0.388**	0.322**	0.199**
Communication (C)	0.354**	0.213**	0.311**	0.291**	0.271**	0.231**	0.270**	0.118	0.321**	0.293**	0.042	0.251**	0.267**	0.318**

Note: scores obtained by child above the diagonal; scores obtained by parent below the diagonal; correlation between scores of the child and parent on the diagonal. Correlation values between the PedsQL 4.0 Generic Core Scales Total Score with the PedsQL 3.0 Cancer Module Scales are set in boldface. Correlation values between the scores of child and parent are underlined. All correlations present significance levels when *p < 0.05 and **p < 0.01 (2-tailed).

Table 8 presents the correlation between scores of the child self- and parent proxy-reports of the Genetic Core Scales and the Caner Module. A positive correlation between child self- and parent proxy-reports was found on all scales of both the Generic Core Scales and the Cancer Module.

## Discussion

This study demonstrated the feasibility, reliability, and validity of the Chinese mandarin version of PedsQL 4.0 Generic Core Scales and 3.0 Cancer Module in children with cancer living in mainland China.

With regard to obtention of result, only a short amount of time was required to complete the questionnaires. This short completion time made these two instruments particularly applicable to the fast-pace setting of an outpatient clinic. Additionally, the overall percentage of missing item responses across the PedsQL scales was low, indicating that children and their parents were able to provide good data regarding the child's HRQOL. However, our results showed that there were several items, i.e. 'worry about side effect from medical treatment' and 'it is had for me to ask the doctors and nurses questions', had a high missing rate. We found that the percentage of missing values was primarily from the 2-7 years old children. The reason may be that some parents regarded their children as too young to understand the questions, or some children aged 5-7 years had difficult to understand these questions, since not all of them attend kindergarten or school. This finding is comparable with previous reports of the PedsQL Cancer Module [9], and also in yline with other PedsQL disease-specific modules [38]. This indicated that some modifications for the items of these subscales in Toddler and Young Child version scale were necessary.

No (for Generic Core Scales) or minimal (for the Cancer Module) floor effects and more accentuated ceiling effects for both scales means that distinction by Chinese mandarin version of the PedsQL 4.0 Generic Core Scale and 3.0 Cancer Module between children who do extremely well or just well is less than excellent [39]. On the other hand, this finding support the opinion that the PedsQL scaling range is acceptable for use in patients experiencing greater health-related problems, which is the area of most concern in research with severe or chronic illness [40,41].

For internal consistency, both the PedsQL 4.0 Generic Core Scales and 3.0 Cancer Module reliabilities approached or exceeded the alpha coefficient standard of 0.7 for most scales. The PedsQL 4.0 Generic Core Scales Total Score for parent proxy-report exceeded an alpha of 0.90, recommended for individual patient analysis, making the Total Scale score suitable as a summary score for the primary analysis of HRQOL outcome in clinical trials and other group comparisons [42]. The total score of the School Functioning, Pain and hurt, Perceived physical appearance, and Communication subscales for child self-report did not approach or exceed 0.70. These findings are consistent with reliability estimates seen in the original English version and the German Version [9,19]. This low internal consistency may be related to the small number of items that compose the subscales and the low level of schooling in the sample. Although Cronbach's alpha represents the lower bound of the reliability of a measurement instrument, and is a conservative estimate of actual reliability [43], scale that did not meet the 0.70 standard should be used only for descriptive analyses.

It is recommended that the interval between measurements must be long enough to reduce the effects of memory and short enough to diminish the likelihood of systemic alterations. Previous studies found that a period of 1 to 4 weeks is considered adequate [44,45]. In this study, we used a 1-3 weeks interval. Meanwhile, patients were selected who were considered to be stable and were not expected to change before completing the questionnaires for the second time. All scales for both the child self- and parent proxy-reports showed good to excellent reliability, indicating that the Chinese mandarin version of PedsQL 4.0 Generic Core Scale and Cancer Module are stable over time.

As can be expected, the PedsQL 4.0 Generic Core Scales indicated better HRQOL in children of the general population than in children with caner on all scales, which support the construct validity of the translated instrument. Additionally, we found that in child self- and parent proxy-report, physical health subscale scores and some psychosocial health subscale scores in children off-treatment over 12 months were significantly higher than children on-treatment in the two instruments. This result was similar to another study assessing HRQOL scores between children on-treatment and those who were off-treatment [33]. Meanwhile, we found that many psychosocial health subscale scores were not significant improved among children who had been treatment over 12 months. This finding is also in line with studies of HRQOL in children with cancer [19,20,33]. It would seem to reflect the long-term burden of psychosocial trials which individuals face as they grow older: they may be afraid, often on an unconscious level, that the disease will recur, they may experience a setback resulting from the stresses during treatment or they can be confronted with new problems evolving from the illness or long-term side effects of treatment [46]. We, like others, believe that psychosocial support remains important long after treatment has completed, and even when the physical health appears well [21,47].

The results of the factor analysis in general support the hypothesized factor structure of the PedsQL. For the PedsQL 4.0 Generic Core Scales, all items split into two different factors. The results do not resemble Varni's five factor structure in the original PedSQL version [41]. In their study, Emotional Functioning items in both child self- and parent reports do not split into two different factors. But the findings of factor analysis may be sample-specific, other studies showed different results too [27,48], this is why the factor structure should be reinvestigated in clinical samples. For the PedsQL Cancer Module, exploratory factor analysis identified 8 factors for child self-reports, replicating those of the original theoretical dimensions. In parent proxy-reports, those items of the Procedural anxiety and Treatment anxiety subscales of the original theoretical dimension loaded on the same factor, reducing the number of factors to 7. In mainland China, parents generally believe that the fear of injection, surgery or other invasive treatments is the main reason why their children try to avoid going to the hospital [49], so that their worry about treatment may linked to the 'Procedural anxiety' factor.

Consistent with the conceptualization of disease-specific symptoms as causal indicators of generic HRQOL, the intercorrelations between the PedsQL 4.0 Generic Core Scales total score and PedsQL 3.0 Cancer Module were in medium to large range, supporting construct validity. Regarding the agreement between child self- and parent proxy-reports, our data showed moderate to good agreement both for the Generic Core Scales and the Cancer Module. Finding higher correlations for the observable parameters in general, like the Physical Functioning Scale in the Generic Core Scale and Pain and hurt, Nausea, and Treatment anxiety in the Cancer Module. This finding is consistent with that of previous studies [33,38]. Although child self-reports is critical, perspectives of parents also are important. In clinical practice, there may be circumstances when the child is too young, or too ill too complete an instrument, and parent proxy-report may be needed in such cases. Additionally, it is typically parents' perceptions of their children's HRQOL and symptoms that influences health care utilization. Therefore, in cases in which pediatric patients are not able to provide self-report, reliable and valid parent proxy-report instrument are needed [50].

This study has several inherent limitations. One limitation of the study was that the study sample was entirely composed of patients seeking medical evaluation or treatment for cancer and cannot be considered representative of the general population of pediatric oncology patients. In mainland China, many children with cancer have refused or abandoned treatment for financial reasons, which is unknown in developed countries and regions [51]. Information about the HRQOL in these patients is still unknown. Second, all the subjects in this study were recruited in two of the largest cities (Shanghai and Chongqing) in China. The majority of our patients came from urban areas instead of rural areas. In fact, more than half of China's population now lives in rural areas where only a few children under 14 years of age who have acute leukemia receive protocol based therapy [23]. Therefore, further studies conducted in rural areas are suggested. Third, information on participants' socioeconomic status was not available. Questions still exist as to whether socioeconomic status is associated with HRQOL in children with cancer in our society [52].

On the other hand, the information from standardized questionnaires provided a wealth of information about the physical and psychosocial status of children with cancer living in mainland China. Moreover, we compared the patients with normal children matched for sex and age, and all subjects were recruited from the same health care catchment areas and assessment were carried out in closely related time periods in both groups. Therefore, the study of these children could provide a more accurate picture of pediatric cancer patients referring to clinical practices, and provide relevant clues for future interventions that promote care and support of children with cancer.

## Conclusion

In summary, our results generally support the feasibility, reliability and validity of the Chinese mandarin version of PedsQL 4.0 Generic Core Scales and the Cancer Module. Further studies should focus on testing responsiveness of the Chinese mandarin version scales in longitudinal studies and in other areas, particularly in rural areas. Studies measuring HRQOL in children who have refused or abandoned treatment are also warranted.

## Competing interests

The authors declare that they have no competing interests.

## Authors' contributions

YJ contributed to the design of the study, the conception and interpretation of the statistical analysis, and drafted the manuscript. SYC conducted the statistical analysis, contributed to the interpretation of data, the drafting of the manuscript. KL, NX, XY contributed the acquisition of data. SZ and XMX contributed to the conception and design of the study, and revised of the manuscript. All authors read and approved the final manuscript.
